# Supplementary material for: Splice-Junction-Based Mapping of Alternative Isoforms in the Human Proteome
Source: Cell Rep. Author manuscript; Available in PMC 2020 Jan 15. (PMC6961840; doi:10.1016/j.celrep.2019.11.026)
Supplement: 2 [file NIHMS1546469-supplement-2.zip › DF1/PXD009737/TestisTrypsin/TestisTrypsin_16_SVIL_RPELCTSHSETPTVDDEEKVDER.pdf]

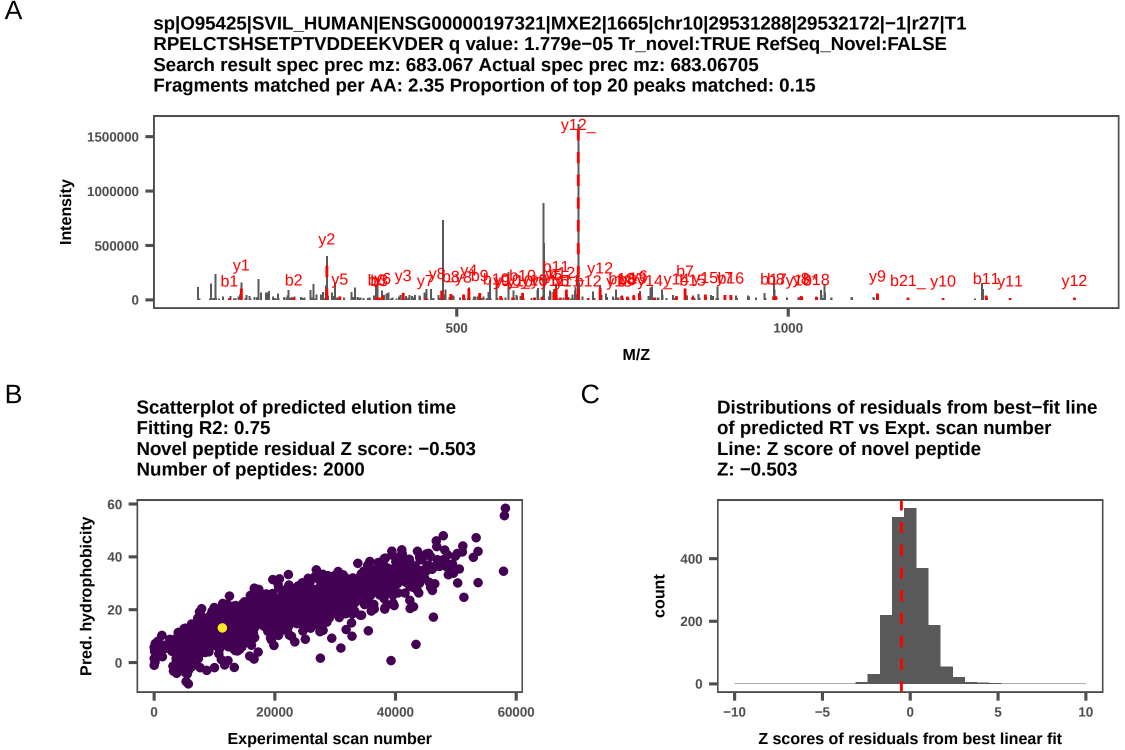
A
sp|O95425|SVIL_HUMAN|ENSG00000197321|MXE2|1665|chr10|29531288|29532172|−1|r27|T1
RPELCTSHSETPTVDDEEKVDER q value: 1.779e−05 Tr_novel:TRUE RefSeq_Novel:FALSE
Search result spec prec mz: 683.067 Actual spec prec mz: 683.06705
Fragments matched per AA: 2.35 Proportion of top 20 peaks matched: 0.15
Intensity
1500000
1000000
500000
0
M/Z
500
1000
y12_
y2
y1
b1
b2
y5
y3
y12
y9
y10
y11
y12
B
Scatterplot of predicted elution time
Fitting R2: 0.75
Novel peptide residual Z score: −0.503
Number of peptides: 2000
Pred. hydrophobicity
60
40
20
0
Experimental scan number
0
20000
40000
60000
C
Distributions of residuals from best−fit line
of predicted RT vs Expt. scan number
Line: Z score of novel peptide
Z: −0.503
count
400
200
0
Z scores of residuals from best linear fit
−10
−5
0
5
10
